# A comparison between stabilization exercises and pelvic floor muscle training in women with pelvic organ prolapse

**DOI:** 10.4274/tjod.74317

**Published:** 2015-03-15

**Authors:** Nuriye Özengin, Necmiye Ün Yıldırım, Bülent Duran

**Affiliations:** 1 Abant İzzet Baysal University, School of Physical Therapy and Rehabilitation, Bolu, Turkey; 2 Yıldırım Beyazıt University Health Science Faculty, Department of Physiotherapy, Ankara, Turkey; 3 Abant İzzet Baysal University Faculty of Medicine, Department of Obstetrics and Gynecology, Bolu, Turkey

**Keywords:** Pelvic organ prolapse, pelvic floor, stabilization exercises, home training

## Abstract

**Objective:**

This study aimed to compare the effectiveness of stabilization exercises and pelvic floor muscle training in women with stage 1 and 2 pelvic organ prolapse.

**Materials and Methods:**

In a total 38 women with pelvic organ prolapse whose average age was 45.60 years, pelvic floor muscles were evaluated with electromyography, and prolapse with pelvic organ prolapse quantification system, and the quality of life with prolapse quality of life questionnaire. Afterwards, the subjects were divided into two groups; stabilization exercise group (n=19) and pelvic floor muscle training group (n=19). Stabilization exercise group were given training for 8 weeks, 3 times a week. Pelvic floor muscle training group were given eight-week home exercises. Each group was assessed before training and after eight weeks.

**Results:**

An increase was found in the pelvic muscle activation response in the 2 groups (p≤0.05). There was no difference in EMG activity values between the groups (p>0.05). A difference was found in the values Aa, Ba and C in subjects of each group (p≤0.05), and the TVL, Ap, Bp and D values of subjects in pelvic floor muscle training group (p≤0.05) in the before and after pelvic organ prolapse quantification system assessment, however, no difference was found between the groups (p≤0.05). A positive difference was found in the effect of prolapse sub parameter in each of the two groups, and in general health perception sub parameter in subjects of stabilization exercise group (p<0.05) in the prolapse quality of life questionnaire.

**Conclusions:**

It was concluded that both training programs increased the pelvic floor muscle strength, provided a decline in prolapse stages. Stabilization exercise has increased general health perception unlike home training, thus, these exercises can be added to the treatment of women with prolapse.

## INTRODUCTION

The American College of Obstetricians and Gynecologists has defined pelvic organ prolapse (POP) as the prolapses of organs in pelvis into the vaginal canal and downwards outside the canal^(1)^. POP includes prolapse of anterior vaginal wall (urethrocele, cystocele), posterior vaginal wall (enterocele, rectocele) and apical segment of the vagina^(2)^. POP is classified under 5 stages as 0, 1, 2, 3, 4 according to its level of severity^(3)^. The possibility of the diagnosis of POP is 50% in women who gave birth once or twice^(2)^. In their study, Slieker-ten Hove et al., notified that there is at least stage 2 POP in 40% of women between the ages 45 and 85 years. In the same study, it has been reported that 10% of women with prolapse had more than one surgical operations for the treatment of prolapse^(4)^.

Women with prolapse have various pelvic floor symptoms. Among these symptoms, pelvic severity, feeling of heaviness in the vagina, swelling coming downwards from the vagina, nodule or protrusion, and lumbar pain are frequently seen. Moreover, symptoms of bladder or intestines or symptoms of sexual function disorder can also be seen frequently. These symptoms can be related to the organ with prolapse or they can be independent from the prolapse^(2)^. Surgical interventions, mechanical support and suggestions on life style, physiotherapy and rehabilitation program^(5)^ are the methods used in the treatment of prolapse. Physiotherapy and rehabilitation approaches consist of exercises to strengthen the pelvic floor muscles, pelvic floor supporting, electric stimulation and biofeedback. The aims of application of physiotherapy and rehabilitation methods are increasing the strength, endurance and support of the pelvic floor muscles, preventing the deterioration of prolapse, helping to decrease the severity and frequency of symptoms caused by prolapse and preventing or delaying the surgery^(2^).

Pelvic floor muscular training is an important physiotherapy and rehabilitation approach in women with POP^(6)^. When the studies on this subject are reviewed it is seen that the exercises for strengthening of pelvic floor muscles are given as home-based exercise programs^(6,7,8,9)^. In the literature, it has been reported that the stabilization exercises are effective on the pelvic floor strength of healthy individuals and other disease groups^(10,11,12,13)^. However, as far as we know, there is no study on the effects of stabilization exercises performed under physiotherapists supervision in clinics on the muscular strength, prolapse stages and life quality of the women with POP. In the light of the existing knowledge, our aim was to compare the effectiveness of stabilization exercises training and pelvic floor muscular training in women with stage 1 and 2 POP.

## MATERIALS AND METHODS

**Figure 1:**
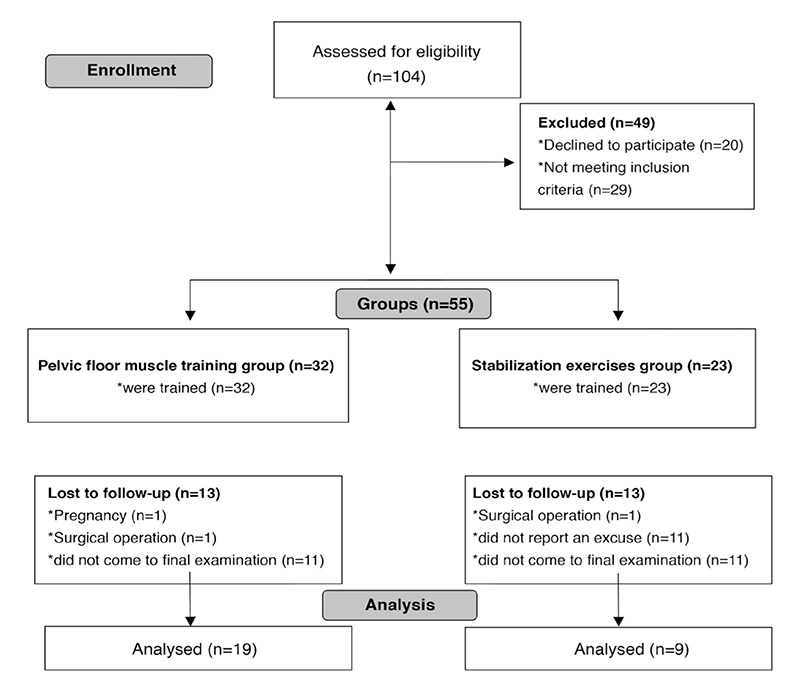
Flow diagram

Fifty-five patients with POP who applied to the outpatient clinic of gynecology and obstetrics department between December 2011 and August 2012 were included in the study (Figure 1). The necessary study approval (B.30.2.ABÜ.0.20.05.04-050.01.04-59) was taken from Abant İzzet Baysal University Ethics Committee and the written consents were taken from the patients.

The patients were at stage 1 and 2 prolapse according to the POP Quantification System (POP-Q) and they have given birth at least one year ago. Exclusion criteria were; POP surgery (4 cases), breast feeding (1 case), pelvic organ cancer (1 case), neurological disease (2 cases), drug treatment for psychological problems (2 cases), untreated urinal infection (1 case), stages 0,3 or 4 according to the POP-Q (10 cases), intending to get pregnant in the following 6 months (1 case) and not being able to contract pelvic floor muscles (8 cases). Capabilities of pelvic floor muscle contraction were detected with the bimanual evaluations. None of the cases in the study has attended at urogynecologic physiotherapy program before.

Muscular strengths, prolapse stage and life qualities of the cases were evaluated before and after the treatment. The evaluation of the cases with POP has been done via using POP-Q created by the International Continence Society, American Urogynecologic Society and the Gynecologist Surgeons Society. The subjects were requested to take lithotomy position. In order to make measurement, speculum and measuring set were used. In the POP-Q evaluation, 9 points Aa, Ba, Ap, Bp, C, D, genital hiatus, perineal body and total vaginal length were used. Hymen point being regarded as zero, the position of six points in anterior (Aa, Ba), superior (C, D) and posterior (Ap, Bp) vagina were measured as “hymenium proximal” (negative number) or “hymenium distal” (positive number) in centimeters (cm). In all other measurements except for the total vaginal length, the patient was strained and the prolapse in each segment was evaluated. POP-Q staging was made after the measurement values were placed in a 3x3 table^(1)^. The muscle activation responds of the pelvic floors of the women were evaluated by using a Myomed 932 branded (ENRAF NONIUS, the Netherlands) EMG Biofeedback device. During the evaluation, surface EMG electrode and vaginal probe electrode were used. The measurement was repeated three times and the total muscle activation respond was recorded as μV^(14)^.

In order to evaluate the life quality of the participants, the prolapse quality of life scale (P-QOL) was used. The Turkish validity and reliability of this scale was made by Seven and Açıkel. P-QOL survey consisted of the following; sub parameters of general health perception (1 question), the effect of prolapse (1 question), the limitations of functions (2 questions), psychical limitations (2 questions), social limitations (3 questions), personal effects (2 questions), emotions (3 questions), sleep/energy (2 questions) and the level of severity (4 questions). The score of each sub parameter was calculated by using different formulas^(15)^. Having a result closer to 0 was evaluated as that the life quality of the participant was good.

After giving information about the treatment methods to the 55 patients, who had the diagnosis of POP and who met the criteria of this study, groups were constructed. To the first group, stabilization exercises (SE) were given as a group training together with a physiotherapist, while pelvic floor muscular exercises (PFME) were given as home-based training program to the other group. The training of the subjects in the stabilization exercise group was started with a one-hour theoretical education. The patients have learned how to use abdominal supporter with stabilizer. All of the stabilization exercises were made by using abdominal supporter. The exercise program consisted of warm-up, stabilization exercises and cooling down periods and exercises were performed 3 days in a week as one-hour programs for 8 weeks. How to contract the pelvic floor muscles was taught to the pelvic floor muscular training group. The subjects were requested to do the exercises as follows; for the fast contacting muscles fibers, contract-relax; for the slowly contracting muscle fibers, slowly contract-counting up to ten-keeping contraction-counting up to ten-slowly relaxing-counting up to ten. The patients started to do exercise as 5 sets and 10 repeats in a day. By increasing the number of sets by 5 each day, they went up to 30 sets a day and continued. Following chart was given to patients so that they did the exercises properly and did not forget doing(14). The cases in the study were told that they should be careful while carrying something heavy or doing something hard. They were also warned to keep their weight stable, to do pelvic floor muscle contraction exercises properly and to be careful about their diets to prevent problems such as constipation.

### Statistical Analysis

The results of the tests made before and after the treatment program were evaluated with the Wilcoxon matched pairs test. In order to detect which training created difference among groups, the Mann-Whitney U test was used. At the analysis of the date, the statistical relevance level was defined as p≤0.05. The statistical processes were made in computer environment.

## RESULTS

**Figure 2:**
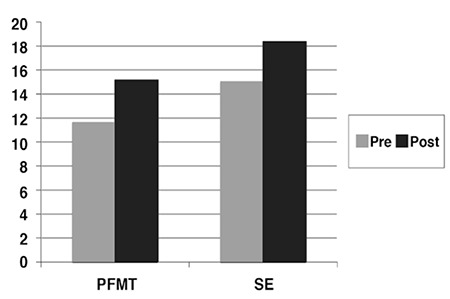
Pre and post EMG activity values of groups

The descriptive statistics of the cases are given in Table 1. The evaluation of the pelvic floor muscle activation EMG respond showed a statistically significant difference (p≤0.05) between the first measurement and the last measurement in patients in the pelvic floor muscular training group (p=0.02) and in the stabilization exercise group (p=0.05) (Figure 2). There was no difference between the groups (p>0.05) (Table 2).

**Figure 3:**
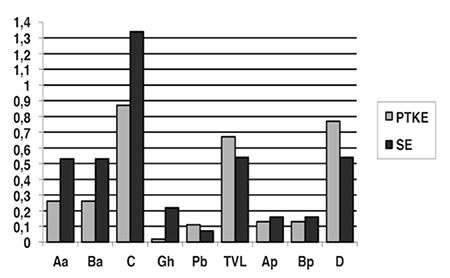
Comparison of POP-Q values between groups

There was a statistically significant difference between the first and last measurement of the Aa, Ba and C values of the cases in both groups (p≤0.05) for POP-Q evaluation. In TVL, Ap, Bp and D values of the pelvic floor muscular training group, a statistically significant difference was detected (p≤0.05). There was a statistically significant difference between the first and the last measurements of the Gh values of the subjects in the stabilization exercise group, (p≤0.05) (Table 3). When we compared the POP-Q measurement values of the pelvic floor muscular training group and the stabilization exercise group, a statistically significant difference was not detected between the groups (p>0.05) (Figure 3). In the analysis made according to the results of the P-QOL, a positive difference (p≤0.05) was detected between the first and the last measurements in the prolapse effect sub parameter of the cases in both groups. In the General Health Perception sub parameter of stabilization exercise group, a positive difference was detected (p≤0.05). In the first and last measurements under the Physical limitations, Personal Effects, Sleep/Energy and Severity level sub parameters of pelvic floor muscular training group, a positive difference was detected (p≤0.05) (Table 4). When we compared the differences in P-QOL scores, a statistically significant difference was detected in favor of stabilization exercise group in the general health perception parameter whereas in personal effects, sleep/energy and severity level sub parameters significant difference was in favor of pelvic floor muscular training group (p≤0.05) (Table 5).

## DISCUSSION

In this study aiming the comparison of the stabilization exercises and pelvic floor muscular training in women with stage 1 and 2 POP, it was shown that both the treatment methods increased the muscular strength, recessed the prolapse level according to the POP symptom score and positively affected some of the sub parameters of quality of life.

It was shown that there were decrease in the cross-sectional area of the levator ani muscle, increase in the genital hiatus, decrease in the muscle strength and increase in the POP severity with pelvic floor dysfunction in women with pelvic organ floor prolapse^(7,16)^. Chen et al. reported that enough pelvic floor muscular strength was important to prevent POP depending on their anterior vaginal wall prolapse (cystocele) bio-mechanic model^(17)^. In this study, the training program aiming to strengthen the pelvic floor muscles was applied since the importance of the pelvic floor muscle strength has been emphasized in the literature. The logical base of the strengthening pelvic floor muscles is to improve the structural support of pelvis via elevating the levator layer to a permanent level inside the pelvis and increasing the stiffness and hypertrophy of the pelvic floor muscles and connective tissues. This enables a more effective motor unit firing (neural adaptation) and increased abdominal pressure and facilitates preventing the prolapse^(18)^. Moreover, strengthening training can lift the pelvic floor and, thus, protrudes the organ up in the cranial direction. Pelvic opening can get tighter and, pelvic organs can stay at their places when abdominal pressure increases^(19)^.

In the literature, there are studies reporting that home-based training program of pelvic floor muscular training given to women with POP increased the muscular strength^(7,8,9)^. In this study, an increase of 3.58 μV was detected in the pelvic floor muscular training group. Based on the result that the women in pelvic floor muscular training group had an increase in their muscular strength although they were not under the observation of a physiotherapist, it was thought that when women could not attend the regular training program with a physiotherapist, it was important to give these home-based exercises to them.

Another method given in the literature for the increase of the pelvic floor muscular strength, is transversus abdominis (TrA) muscle training^(20)^. The basis of this training is pelvic floor muscles and synergistic activity of TrA^(21)^. Culligan et al. reported in their study including 62 women with light to serious pelvic floor dysfunction that there was a need for further studies investigating the use of pilates programs in order to strengthen the pelvic floor muscles and the real use of these exercises in the treatment of the pelvic floor dysfunction^(10)^. When the literature about this topic was reviewed it was seen that exercises via TrA activation increased the muscular strength, but these studies were made on healthy women and women with postpartum urinal incontinence or as home-based programs^(10,11,12,13,22,23)^. Different than the literature, in this study, the women with POP were included in a stabilization exercise group for 8 weeks, 3 days in a week, as 1 hour duration each day together with a physiotherapist. Moreover, pelvic floor muscle contraction was taught to the patients by using stabilizer TrA contraction and intravaginal probe. At the end of the study, it was observed that the stabilization exercises increased the pelvic floor muscle strength by 3.48 μV.

The classification and measurement of POP is difficult. The most commonly used method is clinical classification POP symptom score system^(24,25)^ which is accepted as the golden standard. This study showed that both methods can be used for women with POP since there were significant improvements in Aa, Ba and C values of both treatment groups and there was no difference between the groups. The decrease in genital hiatus opening was only seen in women of the stabilization group because the stabilization exercise training strengthened the muscle, ligament and fascia. Ap and Bp values showed positive changes in pelvic floor muscular training group. There was only one woman in the stabilization training group who had rectocele additionally and this could not create a statistical difference in the group. TVL and D measurements give the total vaginal length when the woman is in normal position. The increase of this length means that uterus is lifted cranially. In both groups, the last measurements were better than the first ones, but it was seen that there was a statistically significant difference in the pelvic floor muscular training group. Since there was no woman with uterovaginal prolapse in the stabilization exercise group, we thought that there might not be any difference.

POP developed by pelvic floor insufficiency and it can lead to clinical conditions which affect the quality of life negatively such as micturition problems including urinal incontinence, defecation problems, problems in sexual life, POP and associated symptoms of, decubitus ulcer in advanced POP, and pelvis pains^(1)^. In their study, Hagen et al. reported that there was enough evidence showing that pelvic floor muscular training is an effective therapy in decreasing the symptoms of prolapse and its cost is suitable therefore it can be given as the first option in the treatment of prolapse^(26)^. Researchers reported that pelvic floor muscular training decreased the mild prolapse symptoms, prevented the progression, healed the pelvic heaviness complaints and increased the quality of life^(8,9,19,27)^. In this study, a positive improvement was detected in both groups in the quality of life sub parameter and these two treatment methods were equal. An increase was detected in general health perception sub parameter of the stabilization exercise group. According to the literature, group therapy has many additional benefits such as peer support, increase of motivation and compliance to the treatment when compared to the individualistic treatments^(28)^. Doing the stabilization exercises as a group treatment and its full body focus explains the increase in the sub parameters.

During formation of the groups, randomization could not be made because the participants were living in different cities and preferences of women regarding the group they would continue were taken into consideration. These can be seen as limitations of this study.

For many pelvic floor diseases, the first treatment option is pelvic floor muscular training because such diseases can be prevented and treated by increasing the pelvic floor muscular strength and coordination. After applying pelvic floor muscular training effectively, protection of the muscular strength and the stabilization exercises for the continuation of the treatment can be offered as life style suggestions to women with pelvic floor dysfunction.

## Figures and Tables

**Table 1:**
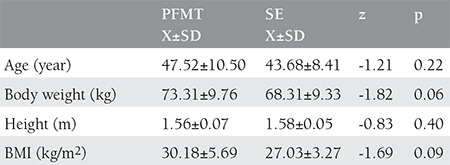
Demographic characteristics of cases

**Table 2:**
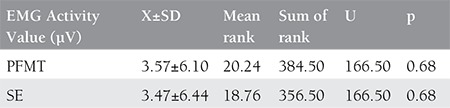
Comparison of EMG activity values between groups

**Table 3:**
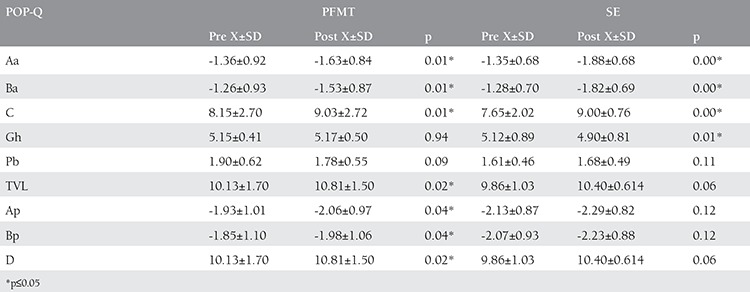
Pre and post POP-Q values of groups

**Table 4:**
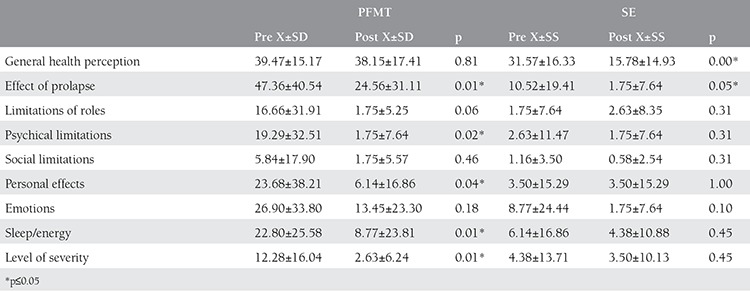
Pre and post P-QOL values of groups

**Table 5:**
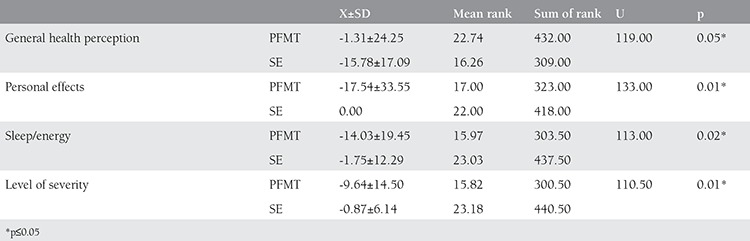
Comparison of P-QOL values between groups
